# Coherent light scattering from a telecom C-band quantum dot

**DOI:** 10.1038/s41467-023-43757-3

**Published:** 2023-12-15

**Authors:** L. Wells, T. Müller, R. M. Stevenson, J. Skiba-Szymanska, D. A. Ritchie, A. J. Shields

**Affiliations:** 1 Toshiba Research Europe Limited, 208 Science Park, Milton Road, Cambridge, CB4 0GZ UK; 2 Cavendish Laboratory, University of Cambridge, JJ Thomson Avenue, Cambridge, CB3 0HE UK

**Keywords:** Quantum dots, Single photons and quantum effects

## Abstract

Quantum networks have the potential to transform secure communication via quantum key distribution and enable novel concepts in distributed quantum computing and sensing. Coherent quantum light generation at telecom wavelengths is fundamental for fibre-based network implementations, but Fourier-limited emission and subnatural linewidth photons have so far only been reported from systems operating in the visible to near-infrared wavelength range. Here, we use InAs/InP quantum dots to demonstrate photons with coherence times much longer than the Fourier limit at telecom wavelength via elastic scattering of excitation laser photons. Further, we show that even the inelastically scattered photons have coherence times within the error bars of the Fourier limit. Finally, we make direct use of the minimal attenuation in fibre for these photons by measuring two-photon interference after 25 km of fibre, demonstrating finite interference visibility for photons emitted about 100,000 excitation cycles apart.

## Abstract (web summary)

Developing quantum networks would require reliable sources of coherent quantum light at telecom wavelengths. Here, the authors employ elastic scattering of excitation laser photons on InAs/InP quantum dots to demonstrate the emission of telecom photons with coherence times longer than the Fourier limit.

## Introduction

Establishing long-distance quantum networks relies on the efficient exchange of quantum information, conveniently encoded in photonic qubits^1–3^. Quantum light sources emitting at telecom wavelengths are fundamental to this endeavour, due to the minimal absorption window of standard fibre networks at these wavelengths. As a consequence, there has been much interest recently in developing novel quantum systems with direct emission at these wavelengths, where III-V semiconductor systems ranging from InP to wide bandgap materials such as GaP and SiN^4–9^ have shown promise as quantum light sources. For emission in the telecom C-band, quantum dot (QD) technology has been most prominent so far^10^, where two different material systems, modified InAs/GaAs and InAs/InP based, respectively, have been pursued^11–18^. These systems have made leaps in their development recently, maturing from showing evidence of single photon emission^19,20^ to demonstrations of entangled photon emission^21,22^ and the development of a spin-photon interface^23^.

A key component for any interference-based quantum network applications is a source of coherent photons. The coherence of the photons is ultimately limited by the radiative linewidth of the underlying transition, where the coherence time *T*_2_ cannot exceed twice the radiative lifetime *T*_1_ (Fourier limit). However, for solid state emitters, reaching this limit is very challenging due to the inevitable coupling of the emitter to its host matrix and the associated decoherence processes. While resonant excitation is key to minimising such noise processes^24^, previous demonstrations of Fourier-limited emission from QDs have been limited to lower-wavelength regions around 900 nm and have further relied on cavity enhancement, reducing *T*_1_ below the timescale of the dephasing processes^25–27^, or on manipulation of the noise processes in the environment^28^.

To produce photons with coherence times even beyond the Fourier limit, it is possible to take advantage of an elastic scattering process often termed Resonant Rayleigh Scattering (RRS), whereby resonant laser light can be elastically scattered from quantum emitters even at excitation powers approaching saturation^29^. In this case, the coherence time of the scattered photons is inherited directly from the driving laser, removing any limit imposed by the transition lifetime. First demonstrated in 900-nm QDs a decade ago^30,31^, this phenomenon continues to be of fundamental interest, and recent work has led to a much-improved understanding of the underlying processes^32,33^. However, the effect has never been observed for telecom wavelength emitters, where arguably it has the highest impact for practical quantum networking applications such as coherent quantum key distribution schemes^34^.

Here, we use the InAs/InP QD platform to demonstrate photons with coherence times much longer than the Fourier limit at telecom wavelength. We first establish resonance fluorescence on a neutral exciton (*X*) transition and characterise the purity of the emission as well as the signal-to-background ratio achievable in our system. Next, we measure the coherence times of resonantly scattered photons in a Michelson interferometer setup, allowing us to directly observe coherence times beyond the Fourier limit due to RRS. The presence of photons with such ultralong coherence times is further evidenced by a distinct signature in the correlation traces of a two-photon interference measurement, recorded in a Hong-Ou-Mandel interferometer. This signature can be modelled analytically to extract the fraction of elastically scattered photons. Finally, we investigate the two-photon interference visibility of photons emitted ~100,000 emission cycles apart by sending one of the two photons through a 25-km fibre spool, enabled by the minimal absorption loss experienced by the QD emission near the telecom C-band.

## Results

### Resonance fluorescence and single photon purity

**Fig. 1:**
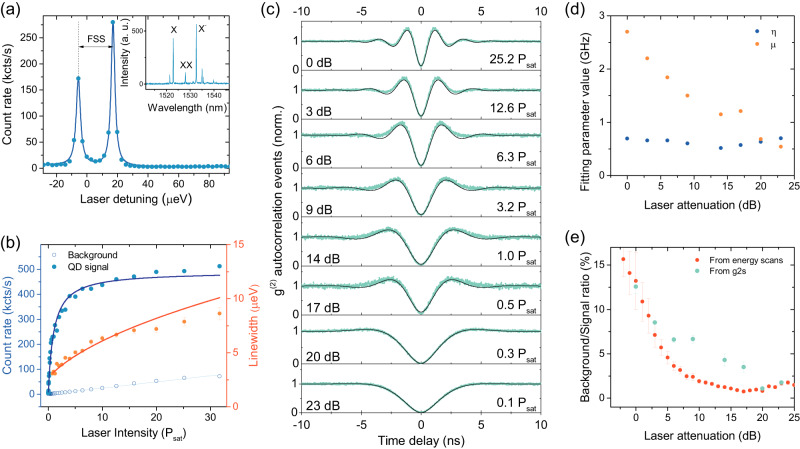
Resonant excitation of a telecom wavelength quantum dot. **a** Absorption spectrum of a neutrally charged exciton, showing two transitions separated by the fine structure splitting (FSS). The full emission spectrum of this QD, recorded under non-resonant excitation, is shown in the inset. **b** Power dependence of the extracted emission count rates (blue filled circles), the laser background count rates (blue open circles) and Lorentzian linewidths (orange circles), all extracted from double Lorenzian fits to power-dependent absorption spectra. To provide meaningful plots and fits, laser attenuation values used in the experiment have been converted to a laser power using the formula *P*/*P*_0_ = 10^−*Att*(*dB*)/10^ and normalised to the saturation power extracted from the fit as described in the text. **c** Autocorrelation data (green circles) and their fits as described in the text (black curves) as a function of laser attenuation. At low attenuations, corresponding to high excitation power values, Rabi oscillations are observed. **d** Values of fitting parameters obtained from the fits in (**c**). Error bars are partly within the symbols. **e** Laser attenuation dependence of the background to signal ratio, extracted from the double Lorentzian fits in (**a**) (red circles) as well as from the autocorrelation measurements shown in (**c**) (cyan circles). Error bars denote the 95% confidence bounds on fitting coefficients.

Our sample consists of InAs/InP quantum dots in a weak planar cavity formed by two Bragg mirrors. For enhanced extraction efficiency, it is further topped by a Zirconia hemisphere. Further details about the sample are given in “Methods”. Under above-band, non-resonant excitation at 850 nm, we record the typical spectrum from such quantum dots^35^ shown in the inset Fig. 1a. To resonantly excite the neutral exciton (*X*) studied here, we tune a narrowband cw laser across the resonance energy and remove any backscattered laser light using polarisation suppression, as further described in “Methods”. Guiding the emission from the quantum dot to superconducting single-photon detectors, we observe two transitions separated by the *X* fine structure splitting, as shown in Fig. 1a. Fitting the absorption spectrum with a double Lorentzian, we determine the fine structure splitting to be of 22.77 ± 0.27 μeV. The relative intensities of the transitions are given by their respective overlap with the excitation and detection polarisation in our system, which was set to allow both transitions to be visible for maximum efficiency.

For the remaining measurements described here, we focus on the higher energy transition. Repeating the wavelength scans of the excitation laser as a function of power, we can extract the maximum emission intensity at each power. This results in the count rate saturation behaviour clearly seen in Fig. 1b (blue data points), with a saturation count rate *I*_*sat*_ of 491 ± 9 kcts/s extracted from a fit to the theoretically expected behaviour, $R={R}_{sat}\frac{P}{P+{P}_{sat}}$. Here, *R* is the recorded count rate, *P* is the excitation power, and *P*_*sat*_ is the saturation power. Fitting the absorption spectra to a Lorentzian lineshape further allows us to extract the linewidth as a function of power, shown by the orange data points in Fig. 1b. The linewidths can be fitted using the square root dependence on laser intensity expected from pure power broadening, which indicates a natural linewidth of 2.7 ± 0.12 μeV. Note that because each data point in the RF scan takes about a second to acquire, this linewidth does not reflect the instantaneous coherence time of photons measured below. Further, background emission at the relevant transition energy (consisting of residual laser light as well as detector dark counts and ambient background contributions) can be extrapolated from an empirical polynomial fit to background data only and shows a linear dependence in laser power (Fig. 1b open circles and cyan curve).

Next, the emission is guided to a Hanbury-Brown and Twiss setup for autocorrelation measurements. These are repeated for excitation powers spanning more than two orders of magnitude, as seen in Fig. 1c. All measurements show a pronounced antibunching dip at zero delay. For higher excitation powers, we further observe the onset of Rabi oscillations, which can be fitted using the expected theoretical description

1 $${g}^{(2)}(\tau )=1-\exp (-\eta | \tau | )\left[\cos \mu | \tau |+\frac{\eta }{\mu }\sin \mu | \tau | \right],$$

where *η* = (1/*T*_1_ + 1/*T*_2_)/2 and $\mu=\sqrt{{\Omega }^{2}+{(\frac{1}{{T}_{1}}-\frac{1}{{T}_{2}})}^{2}}$. The dependence of these fitting parameters on the excitation power is given in Fig. 1d and shows that *η*, which gives the decay of the oscillations, is similar across the different powers, whereas the effective oscillation frequency *μ* is reduced with decreasing power as expected. They encompass the three physical quantities *T*_1_, the excited state lifetime, *T*_2_, the coherence time, and Ω, the Rabi frequency. To determine any two of these, the third one has to be measured independently. In our case, we will measure *T*_2_ to extract *T*_1_ and Ω further below.

From the autocorrelation data at zero delay, we can further extract the background/single photon signal ratio under resonant excitation, as shown in Fig. 1e. Values down to 0.01 ± 0.001 are reached when exciting at 23 dB attenuation. This is about an order of magnitude lower than under non-resonant excitation for these QDs^35^ and comparable to other work resonantly exciting telecom wavelength QDs^36,37^ but does not yet quite reach values reported at lower wavelengths. To investigate where this emission background resulting in non-zero *g*^(2)^(0) values comes from, we compare the *g*^(2)^(0) values to the signal-to-background ratio determined via the Lorentzian fit to the absorption spectrum, which is shown in Fig. 1e as well. This ratio is dominated by residual laser leakage from the excitation laser due to imperfect polarisation suppression at high excitation powers, when the QD transition is saturated, and reaches a minimum of around 19 dB attenuation. For lower powers, detector dark counts and ambient background become significant. At the high as well as the low end of excitation power, the *g*^(2)^(0) values agree very well with the independently determined background ratio, letting us conclude that ambient background and detector dark counts are the biggest contributors to *g*^(2)^(0) values at low excitation powers, while at higher powers excitation laser leakage constitutes a more significant fraction of the total emission.

### Coherence time of resonantly scattered photons

**Fig. 2:**
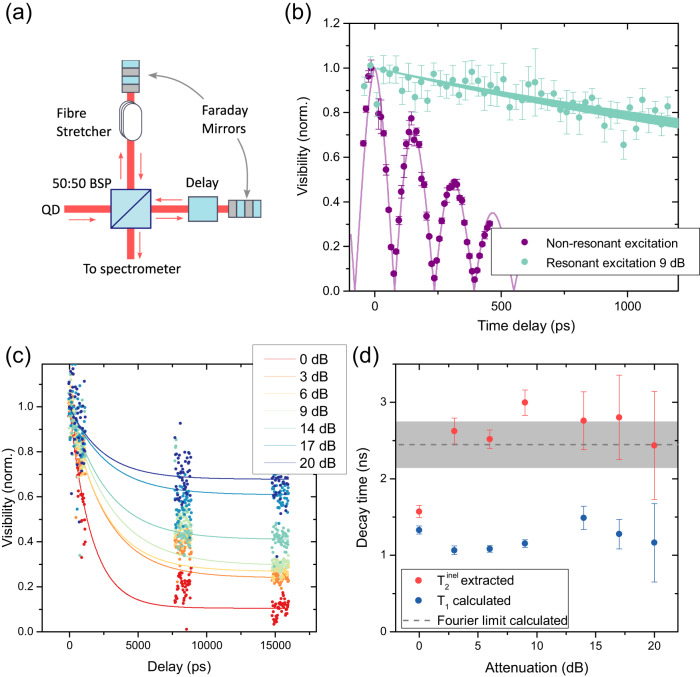
Coherence time of resonantly scattered photons. **a** Michelson interferometer experimental setup. Our fibre-based setup consists of a 50:50 beamsplitter (BSP) to separate QD emission into two arms, where one arm contains a fibre stretcher to record interference fringes and the other arm contains a coarse fibre delay. Faraday mirrors at the end of each arm ensure polarisation matching at the BSP, from where the interference signal is sent to a spectrometer for detection. Interference results were normalised to the visibility achieved by the narrowband cw laser. **b** Visibility of measured interference fringes as a function of the time delay *τ* under non-resonant (purple circles) and resonant (cyan circles) excitation. The solid cyan line represents a fit according to Eq. (2) in the main text, with the shaded area as the error bar of ${T}_{2}^{inel}$. The solid purple line represents a fit to the data as described in the main text. **c** Visibility of interference fringes under resonant excitation, for a range of power levels and for much longer time delays. Filled circles describe measured data points, whereas solid lines describe fits according to Eq. (2) in the main text. **d** ${T}_{2}^{inel}$ times extracted from the initial decays of the signals in (**c**) and calculated *T*_1_ times for all powers. The deduced Fourier limit is indicated by the grey dashed line with the grey shaded area indicating the error bar. Error bars denote the 95% confidence bounds on fitting coefficients.

Resonant excitation is also expected to greatly reduce dephasing compared to non-resonant excitation mechanisms^24^ and increase photon coherence times. To quantify this effect in our QDs, we measure the coherence time of the emitted photons using a fibre-based Michelson interferometer, as schematically shown in Fig. 2a. First, we establish a benchmark and compare this QD to our previous results^17,35^, by measuring the emission coherence time under non-resonant cw excitation at 850 nm. The resulting visibility as a function of time delay between the two arms of the interferometer can be seen in Fig. 2b. It is fitted with the Fourier transform of a double Lorentzian as described in reference^35^, accounting for the interference between the two fine structure split *X* states. From the fit, we can extract a coherence time of 447 ± 15 ps at saturation power. This is comparable to previous best measurements from similar quantum dots^17^ and exceeds the best result reported under non-resonant excitation for modified InAs/GaAs-based telecom wavelength QDs^38^. The observed fine structure splitting of 26.3 ± 0.1 μeV is further close to the value extracted from the resonant scan in Fig. 1a.

Next, we record the interference visibility under resonant excitation, driving the higher energy transition in Fig. 1a. As shown by the cyan circles in Fig. 2b, the visibility of interference fringes now persists for much longer time delays, indicating dramatically reduced dephasing. Note that the beating signal observed under non-resonant excitation is now absent because only one of the two *X* transitions is selectively excited. While a clear slope is visible over the time delays accessible by our fibre delay (1200 ps), to explore the limits of coherence time in the QD emission, we had to extend the total delay in the interferometer by adding extra fibres. Two delays of 1 m each were added to observe interference fringes at delays around 7.5 ns and 15 ns, respectively. We then repeated the measurement for excitation laser intensities spanning more than two orders of magnitude. Figure 2c shows the resulting visibilities. These measurements show that even at very long delays, interference visibilities up to 0.67 ± 0.01 can be observed for low excitation powers. These delays are far beyond the Fourier limit of 2.45 ± 0.30 ns we calculate for this transition further below. We further notice that for most of the measurements, the visibility at delays of 7 ns and 15 ns are very similar. This indicates that the emission from the QDs contains two components: the first component leads to the initial decay of visibility over the timescale of a few ns and is due to inelastically scattered photons with coherence times given by the transition linewidth. This component corresponds to the conventional coherence time *T*_2_ used so far in this manuscript. The second component leads to a power-dependent constant background visibility over the timescales observed. This component is due to elastically scattered RRS photons. Accordingly, we fit the data with a total visibility *V*_*tot*_ determined by the sum of these two components, each described by their respective timescales ${T}_{2}^{inel}$ and ${T}_{2}^{el}$:

2 $${V}_{tot}(\tau )=A{e}^{-\frac{t}{{T}_{2}^{inel}}}+(1-A){e}^{-\frac{t}{{T}_{2}^{el}}}.$$

Here, *A* represents the incoherently scattered fraction of the light, ${T}_{2}^{inel}$ is the coherence time of the inelastically scattered photons and ${T}_{2}^{el}$ is the coherence time of the elastically scattered photons. For the purpose of our fits, ${T}_{2}^{el}$ was set to infinity and the second exponential term therefore set to 1. Looking at Fig. 2c, we can easily identify the two parts to the visibility in the data: there is an initial exponential decay in coherence on the timescale given by ${T}_{2}^{inel}$, followed by a constant section determined by the coherently scattered fraction of the emission. We note that for the highest powers [red and orange data points in Fig. 2c], the data at *τ* ~ 7 ns shows markedly higher visibility than the data at *τ* ~ 15 ns, even though both delays are well beyond the expected Fourier limit and should give similar visibilities originating from only the coherent fraction of the emission. We attribute this to drifts in the experimental setup resulting in a lower effective laser intensity experienced by the quantum dot. To extract the coherent fraction in these cases, we consider the lower possible values by focusing on the data at *τ* ~ 15 ns. Further, as discussed above, there is a non-negligible laser breakthrough in our measurements, especially for higher excitation powers. This breakthrough contribution is estimated from the background in the autocorrelation measurements and the data shown in Fig. 2c is corrected accordingly before fitting the data with Eq. (2).

The coherence time values for inelastically scattered photons, ${T}_{2}^{inel}$, can be extracted from the initial visibility decays similar to the one shown in Fig. 2b for the data recorded at 9-dB attenuation. The resulting values are plotted in Fig. 2d (red data points). These values now allow us to extract the transition lifetime *T*_1_ from the fitting parameter *η* shown in Fig. 1d. The resulting values are given in Fig. 2d as well, and describe the transition lifetime under resonant excitation for the given attenuation levels. Averaging over the obtained *T*_1_ values, we calculate the Fourier limit 2*T*_1_. It is shown as a grey dashed line in Fig. 2d, with the error (grey shaded region) deduced from the standard deviation of *T*_1_ values. For all but the highest excitation power, the coherence times measured for this transition are within the error bars of the Fourier limit determined for the same transition. This means that for resonantly generated single photons from this source, the Fourier limit is actually the lower bound of observed coherence times. Such Fourier-limited emission has previously only been reported around 900 nm^25–28^.

### Signatures of coherent scattering in two-photon interference measurements

**Fig. 3:**
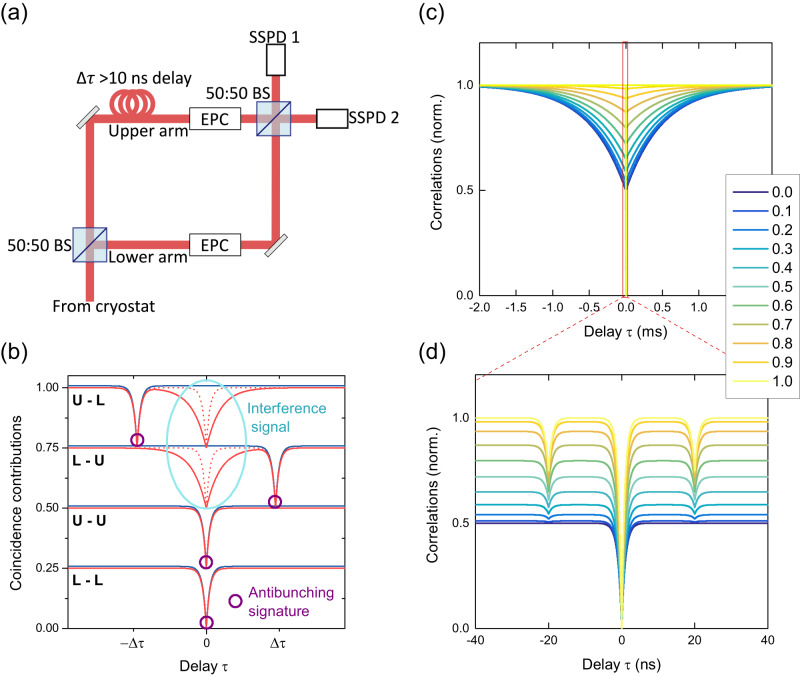
Model of the Hong-Ou-Mandel effect for photons with two coherence times components. **a** Schematic drawing of the HOM setup as described in the text. **b** Schematic showing the individual contributions from the four ways in which coincidences can occur. U (L) indicates the upper (lower) arm was taken for a coincidence event. Blue solid curves stand for cross-polarised coincidences, whereas red solid (dashed) curves stand for co-polarised coincidences where the coherence time was set equal to four times the transition lifetime (exactly the transition lifetime). **c** HOM interference on the timescale of the elastically scattered photon coherence time. The colour scale indicates simulated values for *α* as described in the text. **d** HOM interference on the timescale of the inelastically scattered photon coherence time.

We now investigate the two-photon interference of our QD emission. The collected light is guided to the setup shown in Fig. 3a for Hong-Ou-Mandel (HOM) type measurements^39^. The photons are first separated into two separate arms by a 50:50 beamsplitter and are recombined on a second beamsplitter with an extra delay of ~20 ns introduced in one of the arms. Performing correlation measurements on the outputs on the superconducting single-photon detectors SSPD1 and SSPD2, we measure the degree of two-photon interference on the second beamsplitter. Electronic polarisation controllers (EPCs) are placed in each arm of the setup to control the polarisation of the photons and are set so that the photon polarisation is either fully distinguishable (cross-polarised) or fully indistinguishable (co-polarised).

There are four ways in which coincidences can occur, depending on which of the arms the two detected photons travelled through. The individual contributions are illustrated in Fig. 3b, for the case of a balanced interferometer with equal intensity in both arms. If the two photons detected on SSPD 1 and SSPD 2 both travelled through the same arm (either the upper U or lower L arm in Fig. 3a), an antibunching dip is observed irrespective of the polarisation of the photons, with the width of the dip determined by the natural lifetime of the transition. This is shown by the lower two curves in Fig. 3b. If the photons take different paths at the first beamsplitter, the single photon nature of the emission now manifests itself in antibunching dips at ±Δ*τ*. For co-polarised photons, we measure an additional interference dip at zero delay due to the HOM effect, guiding both incoming photons to the same detector and resulting in an absence of coincidences. The width of this additional dip is determined by the coherence time of the arriving photons, which in general differs from the width of the antibunching dip. The overall coincidence pattern is expected to show a main dip at zero delay, whose depth depends on the relative polarisation of the photons, and two side dips reaching 75% of the total coincidences for a balanced setup.

Mathematically, the co-polarised (∥, *ϕ* = 0) and cross-polarised (⊥, *ϕ* = *π*/2) correlations are given by

3 $${g}_{\phi }^{(2)}(\tau )=\frac{1}{2}{g}^{(2)}(\tau )+\frac{1}{2}\left({g}^{(2)}(\tau+\Delta \tau )+{g}^{(2)}(\tau -\Delta \tau )\right){P}_{HOM}(\tau,\phi ).$$

where Δ*τ* is the delay between photons determined by the HOM setup. Here, *P*_*HOM*_(*τ*, *ϕ*) describes the two-photon interference probability. For cross-polarised light, *P*_*HOM*_(*τ*, ⊥) = 0.5, and for co-polarised light, *P*_*HOM*_(*τ*, ∥) can be calculated from the known functions describing the spatio-temporal modes of the photons at the second beamsplitter in the setup^17,40–42^, as further detailed in “Methods”. The resulting visibility is then defined as

4 $${V}_{HOM}(\tau )=1-\frac{{g}_{\parallel }^{(2)}(\tau )}{{g}_{\perp }^{(2)}(\tau )}.$$

Given that the central dip observed in the HOM measurement is strongly dependent on the coherence time of the photons, we expect a non-trivial signature of RRS photons in the HOM correlations. Intuitively, for photons with coherence times much longer than the delay in the interferometer, one can imagine that the central dip now becomes wide enough to encompass the side dips as well. For fully elastically scattered light, we therefore expect the side dips to vanish completely, effectively reducing the observed correlations to the ones arising from the L-L and U-U contributions in Fig. 3b. For partly RRS photons, the side dips should become shallower.

To express this intuitive understanding mathematically, we analytically calculate Equation (3), modelling the incoming spatio-temporal modes of the field on the second beamsplitter in the HOM setup as the superposition of two travelling waves with coherence times ${T}_{2}^{inel}$ and ${T}_{2}^{el}$ respectively.

5 $${\xi }_{1,2}=\left\{\begin{array}{ll}\frac{1}{N}\left(\alpha \sqrt{\frac{2}{{T}_{2}^{inel}}}{e}^{-\frac{1}{{T}_{2}^{inel}}t-i\omega t}+\beta {e}^{i{\Phi }_{1,2}}\sqrt{\frac{2}{{T}_{2}^{el}}}{e}^{-\frac{1}{{T}_{2}^{el}}t-i\omega t}\right),\quad &t\ge 0\\ 0,\hfill\quad &t \, < \,0\end{array}\right.$$

The subscripts refer to the mode function at input 1 and 2 of the second beamsplitter, respectively, and the phases Φ_1,2_ are random phases included to denote a statistical mixture of the two parts rather than a coherent superposition. The first term refers to spontaneous emission from the QD and has a relatively short coherence time on a nanosecond timescale. This part of the emission has been inelastically scattered during the resonance fluorescence process. The second term denotes photons where the coherence time is inherited from the laser coherence. ∣*α*∣^2^ gives the fraction of inelastically scattered photons in the mode, while ∣*β*∣^2^ = 1 − ∣*α*∣^2^ gives the fraction of elastically scattered photons.

The results for co-polarised photons are shown in Fig. 3c for timescales on the order of the laser coherence time. A broad HOM dip is visible due to the elastically scattered fraction of the emitted photons, with the dip depth decreasing with increasing *α*. The same curves on a nanosecond timescale, corresponding to the QD decoherence timescale, are given in Fig. 3d. If a fraction of the photons have a very long coherence time, it looks as though the dips at ±Δ*τ* are shallow. Furthermore, the apparent side dip depth is dependent on the fraction of photons emitted via spontaneous emission ∣*α*∣^2^. This is the dependency we will use to extract the fraction of elastically scattered photons from our experimental data and normalise our co-polarised data (see “Methods”). Note that for cross-polarised photons, ultralong coherence times have no influence on the expected HOM signal, because it contains no interference terms and is given by the sum of the blue contributions in Fig. 3b.

**Fig. 4:**
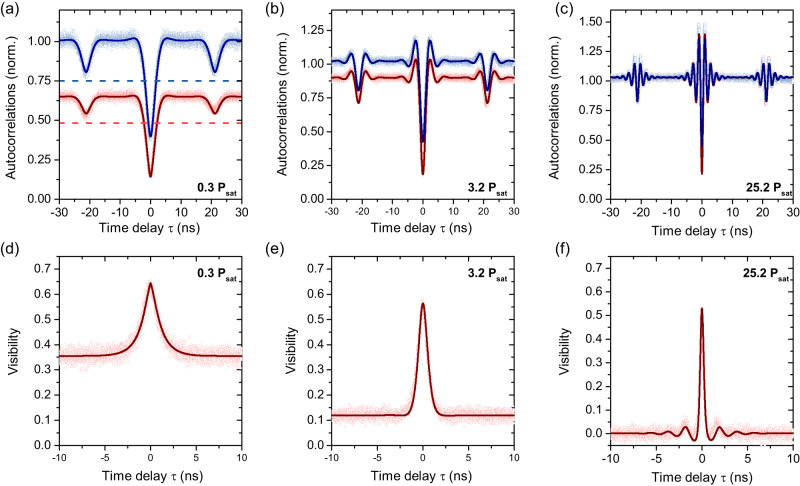
Two-photon interference of a resonantly excited telecom wavelength quantum dot. **a**–**c** Cross- (blue) and co- (red) polarised normalised autocorrelation measurements for HOM with laser power **a** 0.3 *P*_*sat*_, **b** 3.2 *P*_*sat*_ and **c** 25.2 *P*_*sat*_. Corresponding visibility measurements (red circles) with fits (solid curves) are also shown for **d** 0.3 *P*_*sat*_, **e** 3.2 *P*_*sat*_ and **f** 25.2 *P*_*sat*_.

The measured correlations for co-and cross-polarised photons are given in Fig. 4a–c for three different excitation powers. Concentrating on the lowest excitation power as shown in Fig. 4a, the blue data points show a normalised correlation measurement for cross-polarised photons after the HOM setup, where normalisation to Poissonian statistics was performed. This was done by determining average correlation intensities at large delays safely away from any single-photon signatures (~80 ns), where correlations are constant. We observe the expected signature with a centre dip just below 50% and side dips close to the 75% indicated by the blue dashed line. Any reduction of this side dip depth, after deconvolution with detector resolution and correction for imbalanced power in the two arms, is due to residual excitation laser light. Using the measured dip depth to determine the laser background, we find it to be on the order of a few percent for these measurements, in agreement with the direct estimate presented in Fig. 1e.

The same measurement for co-polarised photons is shown in red. Here, as expected from the model described above, the side dips do not reach 75% of the local *g*^(2)^(*τ*) maxima, indicated by the red dashed line. The reason for this reduction in dip depth, which only occurs in the co-polarised data, is the two-photon interference of scattered photons with coherence times much longer than the Δ*τ*. These interference events prevent some of the coincidence events surrounding the side dips, effectively making them appear shallower. In the cross-polarised case, this effect does not occur because the photons are distinguishable by polarisation, and the signature does not contain any interference effects. The origin of these photons is the RSS process, as discussed above, which has a 10-kHz bandwidth in our case. The presence of these ultralong-coherence-time photons affects the normalisation of the co-polarised *g*^(2)^(*τ*), which is defined as two-photon intensity normalised to the product of the time-averaged single photon intensities typically estimated from *g*^(2)^ at ∣*τ*∣ ≫ 0. However, in our measurement, this time average of single photon intensities is underestimated due to interference of the coherently scattered fraction of photons over the entire range of time delays *τ* (up to a few 100 ns) of our measurement. As discussed above and in “Methods”, by estimating this coherently scattered fraction of the emission from the co-polarised side dip depth, we can compensate for this interference effect to correctly normalise our data and determine *g*^(2)^(0) as well as the interference visibility.

To confirm the expected power dependence as given by the RSS mechanism, Fig. 4a–c shows cross- and co-polarised autocorrelation measurements for three different powers. The co-polarised side dips for low powers are decidedly shallower than their cross-polarised counterparts but increase in depth for increasing power, until they match the cross-polarised side dips at high driving powers. At these powers, we further observe oscillations on either side of the antibunching dips. As in Section II, these are a manifestation of Rabi oscillations. The data are fitted using Eqs. (1) and (3). We calculate the resulting visibility using Eq. (1) and present the results in Fig. 4d–f. The maximum visibility values taken from the fits are 0.64 ± 0.09, 0.56 ± 0.05, and 0.53 ± 0.14 respectively. These values are lower than previously reported results at the same wavelength under resonant excitation^37^ and the result may be surprising given the long coherence times and the good two-photon interference visibility results achieved previously under non-resonant excitation^17^. It is important to note however that because of the continuous wave excitation of our source, these maximum visibility values are not a meaningful measurement of the two-photon interference visibility of the emitted photons^40,43–45^, but rather also include the limits of the measurement setup used. For a true measurement of photon indistinguishability, a measurement under pulsed excitation, or an adaptation of ref. ^45^ to include the coherent fraction of the emission in the analysis would be needed, both of which are left for future work.

**Fig. 5:**
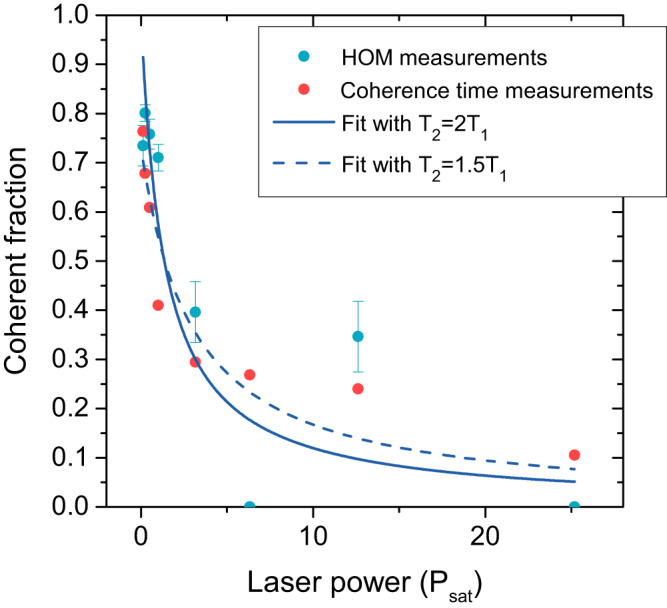
Fraction of elastically scattered photons. Coherent fraction of the emission and fits as described in the main text. Note that for the coherent fraction recorded from HOM measurements (blue data points), there are two measurements with identical dip depth for the co- and cross-polarised cases. These measurements give a coherent fraction of zero, and, as becomes clear from the slope of ∣*β*∣^2^(*D*) in Fig. 7, infinite error bars. These error bars were omitted here for clarity. Error bars denote the 95% confidence bounds on fitting coefficients.

Finally, we plot the values for the fraction of coherently scattered photons as a function of laser intensity, both for the values obtained from the HOM measurements described above and the values obtained from the direct measurements of coherence times presented earlier. As seen in Fig. 5, the two methods give largely agreeing values. Assuming that the laser is in resonance with the transition, theory predicts that the coherent fraction *F*_*CS*_ depends on the driving intensity as follows^46^:

6 $${F}_{CS}=\frac{{T}_{2}^{inel}}{2{T}_{1}}\frac{1}{1+S},$$

where *S* = (Ω^2^*T*_1_*T*_2_) is a generalised saturation parameter. This dependence shows that a ${T}_{2}^{inel}/2{T}_{1}$ value close to 1, as shown here, is critical for observing coherent emission even for low excitation powers where S tends to zero. This explains why elastic scattering has been elusive in other recent works using resonant excitation to drive telecom wavelength quantum dots^23,37^. These works have focused on the strain-relaxed InAs/GaAs system where the observed coherence times are currently still too low^38^.

Fitting our data using Eq 6 and assuming ${T}_{2}^{inel}=2{T}_{1}$, we obtain decent agreement with the experimental results [solid blue curve in Fig. 2d], consistent with the Fourier-limited emission discussed above. However, due to the significant noise in the data, values as low as ${T}_{2}^{inel}=1.5{T}_{1}$ provide equally good fits, as shown by the dashed curve in Fig. 2d. We attribute this noise to imperfect calibration of the HOM interferometer as well as drifts in the setup leading to differing effective excitation intensities or also to phonon sideband contributions, which ultimately limit the coherently scattered fraction of photons^46,47^. While a precise determination of this contribution is left for a future study, the high degree of elastic scattering as well as the ${T}_{2}^{inel}$ times near the Fourier limit suggest that this process is of limited importance in our InAs/InP QDs.

### Two-photon interference visibility of photons separated by 25 km of fibre

Next, we make direct use of the minimal attenuation in fibre at the emission wavelength of our QDs and measure the two-photon interference visibility by adding a 25-km fibre delay to one of the arms of the interferometer shown in Fig. 4a. At 0.173/km dB for the standard SMF-28 Ultra used, this attenuates the signal in the long arm by 4.37 dB or a factor 2.74 compared to the short arm. For comparison, the ~4dB/km-attenuation in specialised fibre at 900 nm would still result in a signal attenuation by 10 orders of magnitude, making such a measurement all but impossible.

**Fig. 6:**
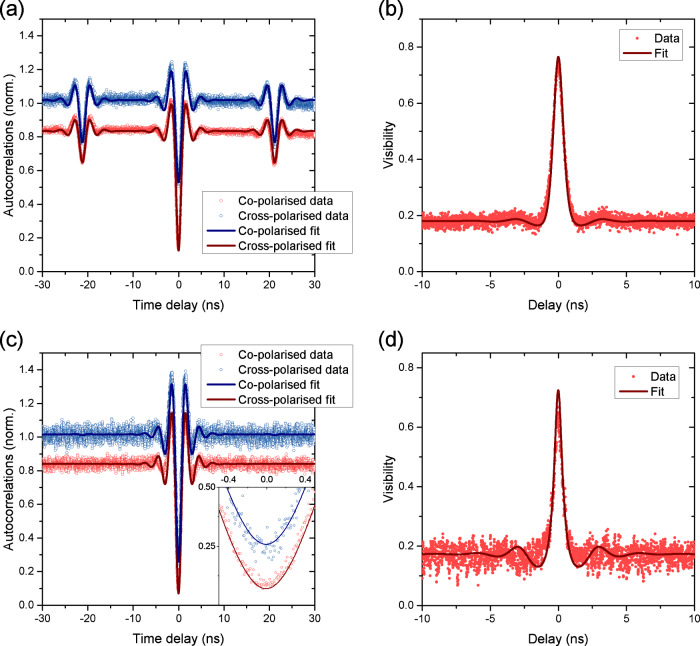
HOM measurements after 25 km of fibre. **a** HOM measurement for dot 2 without the long fibre delay. **b** Visibility calculated from (**a**). **c** Autocorrelation measurements passing one arm of the HOM interferomter through 25 km of fibre. **d** Visibility resulting from (**c**).

For the subsequent measurement, a separate QD, QD 2, was used. To establish a baseline, we first measure the two-photon interference visibility without the extra delay, in the same configuration as above. Figure 6a shows the measured correlations for the co- and cross-polarised cases, where the co-polarised data has been normalised taking into account the coherently scattered fraction of the light as above. The extracted visibility is shown in Fig. 6b. At 0.77 ± 0.06, the maximum is slightly higher than the values measured for QD 1 above, likely due to better experimental overlap of the mode functions. As a further baseline, we also extract the width (1/e) of the interference peak to be 0.95 ± 0.01 ns, which is related to the mutual coherence of two photons emitted ~20 ns apart.

Next, we insert a 25-km fibre spool into the long arm of the interferometer. The measured relative attenuation of this arm is 5.8 dB and includes the effect of extra connectors as well as a switch for polarisation calibration. The correlation measurements resulting from this configuration are shown in Fig. 6c. It is immediately obvious that the side dips seen in the short-delay configuration have disappeared, as they have shifted out of our measurement window to a time delay of ~95 μs, corresponding to the extra distance travelled in the fibre. We can therefore no longer use the measured dip depth of the side dips to extract the coherent fraction and have to rely instead on the short-delay measurement performed at a similar excitation power. We further note that the cross-polarised correlation curve at zero-delay dips well below the 50% mark expected for a balanced interferometer, seen in the inset to Fig. 6c showing a zoom around the zero-delay dips. This is due to the extra attenuation in one of the two arms, which makes correlations arising from two photons having both travelled the short arm dominant compared to the other contributions in Fig. 2b. The expected cross-polarised dip depth is given by ${g}_{cross}^{(2)}(0)=\frac{{T}_{U}^{2}+{T}_{L}^{2}}{{T}_{U}^{2}+{T}_{L}^{2}+2{T}_{U}{T}_{L}}$, where *T*_*U*_ and *T*_*L*_ refer to the transmissions through the upper and lower arm in Fig. 3b, respectively. For our measured attenuation values, ${g}_{cross}^{(2)}(0)=0.33\pm 0.03$, in reasonable agreement with the fitted value of 0.27 ± 0.02. The resulting visibility is shown in Fig. 6d, and we obtain a value of 0.72 ± 0.15. The extra fibre therefore results in a drop in visibility by about 6.5%. More importantly, the width of the interference peak (again 1/e) is now 0.72 ± 0.01 ns, which corresponds to a decrease of 24% compared to the case without the fibre. This means that even after ~100,000 excitation cycles, the transition remains relatively little affected by additional spectral wandering and other slow dephasing processes, demonstrating the stability of our QD transition on the timescale of 95 μs.

## Discussion

We have used HOM measurements as well as direct measurements of emission coherence to show that, except for very high driving powers, the coherence time of scattered photons is at least equal to the Fourier limit, and exceeds it considerably for low driving powers due to a large fraction of coherently scattered photons. For this particular QD, the Fourier limit for inelastically scattered photons is reached even without any additional Purcell enhancement or active feedback on the quantum dot. We further measure the two-photon interference visibility of photons emitted ~95 μs apart by guiding one of the photons through a 25-km fibre spool, a measurement infeasible with quantum emitters at lower wavelength.

An outstanding challenge hampering network integration of C-band quantum dots is further the improvement of the limited extraction efficiency. Recent proposals integrating QD sources into circular Bragg gratings or micropillars structures in the telecom C-band show that coupling efficiencies into single-mode fibres up to around 80% are possible while at the same time also providing Purcell enhancement of factors 10-43^48,49^. Indeed, the first experimental demonstrations of such devices have recently been reported^50,51^. Seeing that under non-resonant excitation the investigated QD has a coherence time only about a factor three higher than the average in InAs/InP QDs^17^, we expect that combining resonant excitation with appropriate photonic engineering will enable the majority of the QDs to perform at the Fourier level with high efficiency. Such a device will be a desirable hardware component for quantum network applications ranging from simple point-to-point quantum key distribution to distributed quantum computation tasks based on interference of entangled photons linking remote locations.

## Methods

### Sample and setup

Our sample consists of a single layer of self-assembled, droplet epitaxy InAs quantum dots, grown in an InP matrix in the centre of a planar cavity. The cavity is asymmetrical, with 20 bottom DBR (distributed Bragg reflector) pairs and 3 top DBR pairs. This enhances the efficiency of the structure by directing emission away from the substrate. A 1-mm diameter, cubic zirconia solid immersion lens (SIL) is attached to the top of the sample via 290 nm of HSQ. This enhances the collected emission intensity by about a factor of 2, as characterised by comparing a small number of quantum dots next to the SIL to those underneath.

The sample is investigated using a confocal microscope with two excitation lasers: a weak non-resonant laser (at 850 nm) to measure photoluminescence from the quantum dot, and a narrow-linewidth (10-kHz instantaneous linewidth as specified by the manufacturer), wavelength-tuneable continuous wave laser scanned around 1532.2 nm to resonantly excite the QD exciton transition. The resonant laser passes through a linear polariser aligned at approximately 45° with respect to the *X* dipole transitions, which in turn are 90° apart. This achieves the best balance between high count rates and good laser coupling. The lasers are focused onto the cooled QD-SIL system within a cryostat kept at 10 K. A quarter wave plate and linear polariser are placed in the detection path to allow for cross-polarisation filtering of the resonant laser, and a grating filter with a width ~0.5 nm is used reject the non-resonant laser. The collected photons then pass through either an HBT setup or a HOM setup (branching ratio of combining 50:50 BSP: 49.8%/50.2%), before being recorded by two SSPDs (superconducting single-photon detectors, efficiency ~90%, individual detector jitter timing resolution ~40 ps, measured timing resolution of the correlation setup 61.4 ± 0.2 ps). Correlation measurements were performed using a time-correlated single photon counter in ‘Histogram’ mode, where the interval between the arrival of a photon on the ‘start’ detector and the nearest click on the ‘stop’ detector is recorded, after which the clock is reset. This mode leads to a prioritisation of coincidences at short delays, leading to the slight slope in coincidences measured in Fig. 6. An individual exciton transition was used for all the data presented here.

### Normalisation of HOM data

**Fig. 7:**
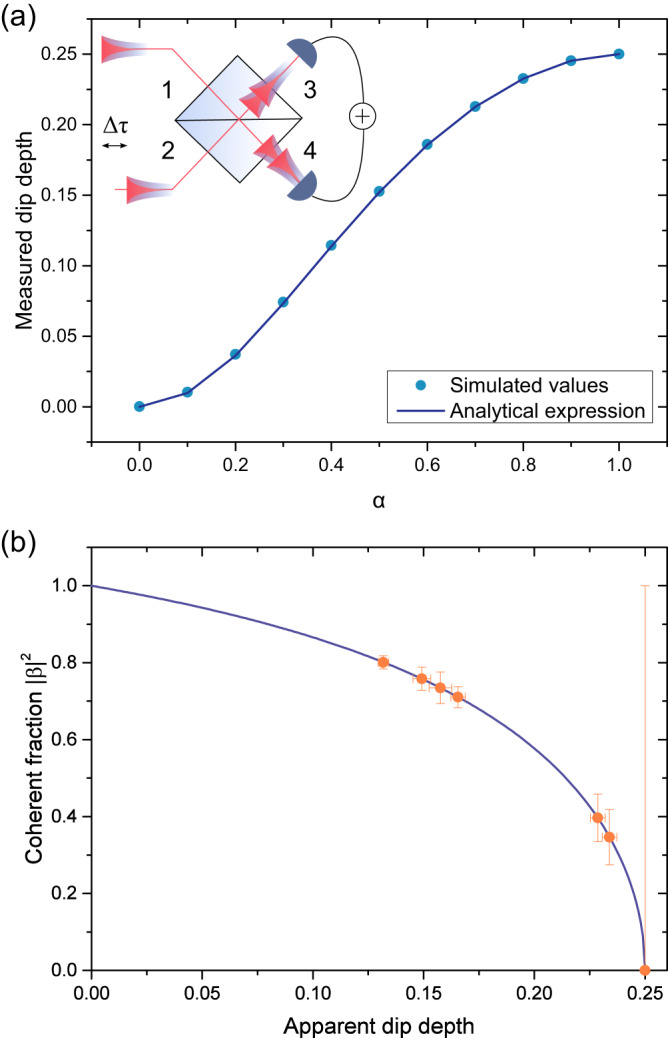
Extracting the coherent fraction from the measured dip depth. **a** Measured dip depth calculated analytically from the model wavefunctions given in Eq. (5). The inset shows a schematic of the second beamsplitter in the HOM setup. **b** Theoretical expression for the coherent fraction ∣*β*∣^2^ as a function of dip depth (blue curve), and coherent fractions extracted from experimentally determined dip depths (orange data points).

To derive an analytical expression for the relationship between the incoherently scattered part of the mode function *α* and the observed dip depth, we explicitly calculate *P*_*HOM*_(*τ*, ∥) using the mode functions given in Eq. (5) for inputs 1 and 2 at the second beamsplitter, as shown in the inset to Fig. 7. Following refs. ^17,40,41^, the probability of a joint click of the two detectors for indistinguishable light is given by

7 $${P}_{\parallel }^{joint}(t,\tau,\Delta \tau )=\frac{1}{4}{\left|{\xi }_{1}(t+\tau -\Delta \tau ){\xi }_{2}(t)-{\xi }_{1}(t-\Delta \tau ){\xi }_{2}(t+\tau )\right|}^{2},$$

where *τ* gives the time delay between the clicks and Δ*τ* accounts for the different arrival times of the photons at the beamsplitter. Integrating over the absolute time *t* and Δ*τ* gives

8 $${P}_{HOM}(\tau,\parallel )=\int\nolimits_{-\infty }^{+\infty }\int\nolimits_{-\infty }^{+\infty }{P}_{\parallel }^{joint}(t,\tau,\Delta \tau )\,{{{{{{{\rm{d}}}}}}}}t\,{{{{{{{\rm{d}}}}}}}}\Delta \tau$$

9 $$=\frac{1}{2}\left(1+| \alpha {| }^{4}{e}^{-\frac{2}{{T}_{2}^{inel}}| \tau | }+2| \alpha {| }^{2}| \beta {| }^{2}{e}^{-\left(\frac{1}{{T}_{2}^{inel}}+\frac{1}{{T}_{2}^{el}}\left.\right)\right)| \tau | }+| \beta {| }^{4}{e}^{-\frac{2}{{T}_{2}^{el}}| \tau | }\right).$$

Entering this expression into Eq. (3), we can calculate the expected correlations, which are shown in Fig. 3. From these curves, we numerically extract the measured dip depth as a function of *α*, shown as blue circles in Fig. 7. We also calculate the measured dip depth *D* analytically, from the parts of Eq. (9) which contribute to the measured dip, *C* = ∣*α*∣^4^ + 2∣*α*∣^2^∣*β*∣^2^, and the parts which do not, *NC* = ∣*β*∣^4^. We obtain the analytical expression

10 $$D=\frac{1}{2}\frac{2| \alpha {| }^{2}-| \alpha {| }^{4}}{2| \alpha {| }^{2}-| \alpha {| }^{4}+1},$$

shown as the blue curve in Fig. 7a. This expression results in a quadratic equation to determine ∣*α*∣^2^ from a measured *D*:

11 $$| \alpha {| }^{2}=1-\frac{\sqrt{8{D}^{2}-6D+1}}{1-2D}.$$

We obtain *D* from the co- and cross-polarised correlation measurements after deconvolution with the detector response and fitting with Eq. (3). To account for laser background and imbalanced intensities in the interferometer, the co-polarised dip depth is normalised to the cross-polarised dip depth. The measured *D* then determines the coherent fraction ∣*β*∣^2^ = 1 − ∣*α*∣^2^. The theoretical expression for ∣*β*∣^2^ as well as the experimentally determined dip depths and extracted coherent fractions are shown in Fig. 7b.

To re-normalise the data, we scale the measured dip depth to the theoretically expected dip depth based on the calculated coherent and incoherent fractions. Given that *NC* + *C* = 1 by construction, the expected dip depth for a given coherent fraction ∣*β*∣^2^ is (1 − ∣*β*∣^2^) × *D*_*cross*_, where *D*_*cross*_ is the cross-polarised dip depth. *D*_*cross*_ = 0.25 in the ideal case of no background emission and a balanced interferometer, and in our experiments without the long delay is found to be *D*_*cross*_ = 0.237 ± 0.003. We therefore apply the following normalisation factor to the co-polarised data:

12 $$N=\frac{(1-| \beta {| }^{2}){D}_{cross}}{D}.$$

This factor is in addition to the Poissonian normalisation applied to the cross-polarised data.

## Data Availability

The authors declare that the data supporting the findings of this study are available from the corresponding author on request.

## Code availability

Code used for the normalisation of the data and calculations for the theoretical model are available from the corresponding author on request.
